# Parameter optimization for stable clustering using FlowSOM: a case study from CyTOF

**DOI:** 10.3389/fimmu.2024.1414400

**Published:** 2024-10-09

**Authors:** Weiyang Tao, Anirban Sinha, Khadir Raddassi, Aridaman Pandit

**Affiliations:** Immunology Discovery Research, AbbVie Cambridge Research Center, Cambridge, MA, United States

**Keywords:** cyTOF, high-dimensional dataset, clustering, FlowSOM, parameter optimization

## Abstract

High-dimensional cell phenotyping is a powerful tool to study molecular and cellular changes in health and diseases. CyTOF enables high-dimensional cell phenotyping using tens of surface and intra-cellular markers. To utilize the full potential of CyTOF, we need advanced clustering and machine learning methodologies to enable automated gating of the complex data. Here we show that critical modifications to a machine learning based FlowSOM package and precise parameter optimization can enable us to reliably analyze the complex CyTOF data. We show the impact of key parameters on clustering outcomes while addressing bugs within the publicly available package. We modified the FlowSOM pipeline to fix the bugs, enable scalability to handle large datasets and perform parameter optimization. We further validated this modified pipeline on a substantial external immunological dataset demonstrating the need of data-specific tailored parameter optimization to ensure reliable definition and interrogation of immune cell populations associated with immune disorders.

## Graphical Abstract

Optimized FlowSOM pipeline for reliable clustering of high-dimensional cytometry data.

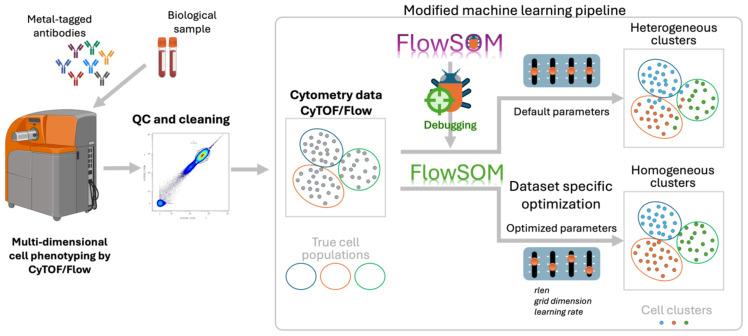

## 1 Introduction

CyTOF (Cytometry by Time of Flight) is a cutting-edge technology in cytometry that utilizes mass spectrometry to measure cellular markers, enabling the simultaneous detection of up to ~100 proteins for hundreds of thousands of cells in a single experiment (1). Unlike traditional flow cytometry, CyTOF overcomes limitations related to spectral overlap, fluorescence spillover and autofluorescence, providing high-resolution and high-throughput analysis of individual cells (2, 3). Its ability to measure a vast number of markers with minimal signal interference and superior sensitivity makes CyTOF a very important technology in multi-dimensional cell phenotyping. By offering a comprehensive view of cell populations and their functional states, it has become an invaluable tool in various research areas, including immunology, oncology, and systems biology, driving advances in understanding cellular heterogeneity and identifying rare cell subsets with unparalleled precision (4–6).

Analyzing complex CyTOF datasets is challenging due to the high dimensionality and inherent noise in the data. As CyTOF can measure multiple markers simultaneously on individual cells, the resulting datasets are large and complex, making it difficult to visualize and interpret the underlying cellular heterogeneity using conventional gating approaches. Moreover, the data may contain technical variations, batch effects, and experimental noise. To unravel meaningful information from such intricate datasets and identify biologically relevant cell subsets, robust clustering algorithms are essential (7). These algorithms should be able to handle high-dimensional data, be resistant to noise and technical variations, and provide reliable and reproducible cell grouping to facilitate downstream analyses and biological interpretation (8). CyTOF data analysis has transitioned from conventional manual gating to sophisticated clustering algorithms (9–11). Although widely used, manual gating is labor-intensive, subjective, and prone to missing rare cell populations and intricate relationships. Advanced clustering algorithms, including PhenoGraph (12), viSNE (13), SPADE (14), and notably FlowSOM (15), have emerged to address these challenges. FlowSOM stands out in the list as a highly relevant method for analyzing high-dimensional biological data, primarily due to its exceptional capacity to automate and streamline the clustering process (16). FlowSOM efficiently conducts clustering, yielding standardized and reproducible analyses with superior accuracy and speed that outperforms other algorithms (17). Notably visualization techniques, such as minimum spanning tree etc., implemented in FlowSOM play a pivotal role in data interpretation. FlowSOM profoundly influences the analysis and understanding of data, particularly in immunology research, aiding in the identification, characterization, and study of immune cell populations and their responses (18–21).

Within FlowSOM, an unsupervised machine learning technique is applied to conduct clustering for high-dimensional cytometry data. In essence, FlowSOM follows the principles of Self-Organizing Maps (SOM) (22, 23) which is an artificial neural network used for unsupervised learning tasks, such as clustering, encompassing three main stages: grid node initialization, training, and termination. Initialization entails a random distribution of nodes across the feature space. The training process calculates the distance between each cell to each node in the feature space, followed by positional updates of nodes through the “attraction” of nearby data points. The termination is performed after the nodes have updated for a certain number of times that is determined by the number of iterations (rlen) and the total number of cells that are used for training.

However, the clustering results obtained from FlowSOM may vary based on several key parameters that influence the algorithm’s behavior. One such parameter is “rlen,” which determines the number of iterations to build the SOM during training. Varying “rlen” can impact the stability of the clusters, with lower “rlen” values potentially leading to an unstable SOM, which may result in unreliable cell clusters. Additionally, the “grid dimension” parameter, which defines the number of nodes in the SOM grid, can influence the granularity of the clustering output. Larger grid dimensions may capture finer details in the data but can also increase computational complexity. Another crucial parameter is the learning rate of the training SOM, a low learning rate may lead to a slow convergence, which, given certain “rlen” value, may result in unreliable cell clusters as well. Therefore, careful optimization of these parameters is vital to obtain consistent and biologically meaningful clustering results with FlowSOM. However, while these parameters could influence FlowSOM output, little attention has been given to parameter optimization to date. This is because most studies utilize the default parameters in FlowSOM, which may not be optimal for all datasets.

Here, we evaluate the impact of varying “rlen” on the quality of CyTOF data clustering using FlowSOM, through which we identified and addressed two bugs in the algorithm. With the bug-free version of FlowSOM, this study systematically explores parameters such as “rlen” and “grid dimension” to elucidate their impact on the stability, reproducibility, and reliability of clustering results for different sizes of CyTOF datasets. This research seeks to highlight the importance of optimizing parameters for clustering to enhance our comprehension of cellular heterogeneity and phenotypic diversity within high-dimensional CyTOF datasets. This allows for a better interpretation of data and clear identification of immune cell population with implication in immune disorders.

## 2 Methods

### 2.1 Data acquisition and preprocessing

We systematically screened the FlowRepository database for large, high-quality CyTOF datasets (MIFlowCyt score > 0.5) uploaded within the last 5 years. The largest multidimensional CyTOF dataset meeting these criteria was utilized for our work which has been previously published ^1^ (24). The dataset comprises of 126,873,075 cells, distributed in 779 human peripheral blood mononuclear cell (PBMC) samples that were collected from 120 intent-to-treat participants, divided into three different groups (Peanut stimulated, Unstimulated, and PMA/Ionomycin-stimulated). Samples were collected at Baseline, week 104, and week 117 time points. After removing the non-marker channels, each sample encompasses 39 marker channels, including markers such as CD3, CD4, CD8, CD19, CD56, TCRgd, and more. Quality checks were executed using pseudo-color plots with “191Ir_dna1”, “193Ir_dna2”, and “115In_LiveDead” channels, employed for filtering cell debris, doublets, and dead cells.

Additionally, to assess the impact of the number of cells on clustering reliability, we derived four smaller datasets by random down sampling from the original dataset using “sample” function without replacement from R base package, resulting in subsets of 27.5 million cells ( $\frac{2^{30}}{39}$ or 27,531,842 cells to be exact), 1 million cells, 100,000 cells, and 10,000 cells (**Table 1**).

**Table 1:** The parameters that are used for optimization.

Parameter	Explanation	Values
Dataset size	The numbers of cells are varied in different datasets. This is evaluated by down sampling the full dataset.	126,873,075 (the full dataset), 27,531,842 (i.e., $\frac{2^{30}}{39}$ ), 10^6^, 10^5^, and 10^4^ cells.
rlen	The number of epochs to train the whole dataset. The product of rlen and the total number cells in the dataset is the total iteration of the grid nodes in SOM. The default and frequently used rlen value is 10.	1, 5, 10, 20, 40, 60, 80, 100, 120, 140, 160, 180, 200, 220, 240, 260, 280, 300, 320, 340, 360, 380, 400, 600, 800, and 1000.
xdim	The width of the grid in SOM. The default is 10, meaning that the number of columns of the node grid is 10.	10, 12, 14, and 16. The value is set as the same as ydim.
ydim	The height of the grid in SOM. The default is 10, meaning that the number of rows of the node grid is 10.	10, 12, 14, and 16. The value is set as the same as xdim. The grid dimension in this article is the combination of xdim and ydim and is denoted as xdim × ydim.
alpha	A vector of start and end learning rates. The default is (0.05, 0.01), meaning that the start learning rate is 0.05, and it is gradually decayed to 0.01 at the end of iteration during the SOM training.	(0.05, 0.001), (0.01, 0.001), (0.1, 0.05), and (0.05, 0.01).

### 2.2 Pipeline for analysis

In the analysis conducted under the R platform (version 4.2.3), we utilized the FlowSOM R package (version 2.6.0) independently, along with our own debugged version (see section 3.1 for more details). Flow Cytometry Standard (FCS) files were initially read using the ‘read.flowSet’ function using the flowCore package (version 2.10.0). Subsequently, the data was transformed using the arcsinh method with a cofactor of 5. A FlowSOM object was created using the ‘ReadInput’ function from FlowSOM with the parameter ‘scale = TRUE.’ Following this, a Self-Organizing Map (SOM) was constructed, varying dimensions (dim = 10, 12, 14, and 16) of a square grid and numbers of iterations (rlen = 1, 5, 10, 20, 40, 60, 80, 100, 120, 140, 160, 180, 200, 220, 240, 260, 280, 300, 320, 340, 360, 380, 400, 600, 800, and 1000) across all cells for the four distinct sub-datasets mentioned above. FlowSOM involves two learning rates (alpha) for the nodes in the SOM grid during training, with the first element in alpha denoting the learning rate for the start of iteration, and the second for the end of iteration. To assess the impact of learning rates, we employed four different combinations: (0.05, 0.001), (0.01, 0.001), (0.1, 0.05), and the default alpha (0.05, 0.01), on the dataset consisting of 1 million cells.

### 2.3 Metrics for the evaluation of reliability and stability of clusters

In this study, we used three metrices including Average Distance to the nearest node, Average Maximum Jaccard Index, and Percentage of Maximum Jaccard Index Over a Threshold to evaluate the reliability and stability of clusters during SOM training.

Average Distance (AD) to the nearest node is defined as


$$\text{AD}=\;\sum_{\text{i}=1}^{\text{n}}\frac{{\text{D}}_{\text{i}}}{\text{n}}$$

where n is the number of cells used for training the SOM, and D_i_ is the Euclidian distance between the i^th^ cell and centroid of its closest node in the SOM after training. The smaller the AD value, the better the SOM nodes represent the clusters in the data. For each run (rlen = 1, 5, 10, 20, 40, 60, 80, 100, 120, 140, 160, 180, 200, 220, 240, 260, 280, 300, 320, 340, 360, 380, 400, 600, 800, and 1000), the AD was calculated for monitoring the updates and performance of the nodes in the SOM. Theoretically, when the SOM is stabilizing and closely representing the clusters in the data, the curve of AD would gradually approach a relatively stable low point and slightly oscillates around the minimum value with the increase of rlen.

Average Maximum Jaccard Index (AMJI), representing the overall similarity of the SOMs in two runs, is defined as


$$\text{AMJI}=\;\sum_{\text{i}=1}^{\text{k}}\frac{\text{max}\left({\text{J}}_{\text{i},.}\right)}{\text{k}}$$

where J is a matrix of Jaccard indices between nodes in the first run N and nodes in the second run M, when comparing the similarity of nodes from two different runs. k is the product of two dimensions of the SOM grid. The Jaccard index between i^th^ node of the first run (N_i_) and j^th^ node of the second run (M_j_) represents a statistical metric to measure the degree of overlap (similarity or diversity) between the cells from the two nodes, and is defined as


$${\text{J}}_{\text{i},\text{j}}=\frac{\left|{\text{N}}_{\text{i}}\cap {\text{M}}_{\text{j}}\right|}{\left|{\text{N}}_{\text{i}}\cup {\text{M}}_{\text{j}}\right|}$$

If the cells in the N_i_ node are exactly the same as the cells in the M_j_ node, then the two nodes from two different runs are exactly the same, i.e. J_i,j_ = 1. On the contrary, if none of the cells in the N_i_ node is present in the M_j_ node, then the two nodes from two different runs are absolutely different, i.e. J_i,j_ = 0. By pairwise computing the Jaccard index of the nodes from two consecutive runs, a matrix of Jaccard indices can be obtained to compare the similarity of nodes in these runs. AMJI is a metric summarizing the Jaccard index of all the nodes from two different runs. Theoretically, the curve of AMJI would gradually approach a plateau and slightly oscillates around the maximum value over the increase of rlen, providing another metric to monitor the stability of the SOM while training.

Percentage of Maximum Jaccard Index Over a Threshold (PMJIOT) is defined as:


$$\text{PMJIOT}=\frac{\sum_{\text{i}=1}^{\text{k}}\left(\text{I}\left(\left\{\text{x∈}{\text{J}}_{\text{i},.}|\text{x}>\text{t}\right\}\right)\right)}{\text{k}}\times 100\%,$$

where t is the threshold, which is either 0.5 or 0.7 in the analyses for assessing the stability of SOM. I is the indicator function, which is defined as $\text{I}\left(\text{A}\right)=\{\begin{array}{c} 1\\ 0 \end{array}\begin{array}{c} ,\text{ if A is true }\\ ,\text{ if A is false} \end{array}$ .

## 3 Results

### 3.1 Debugging FlowSOM package in the context of large datasets and extended rlens

**Figure 1:**
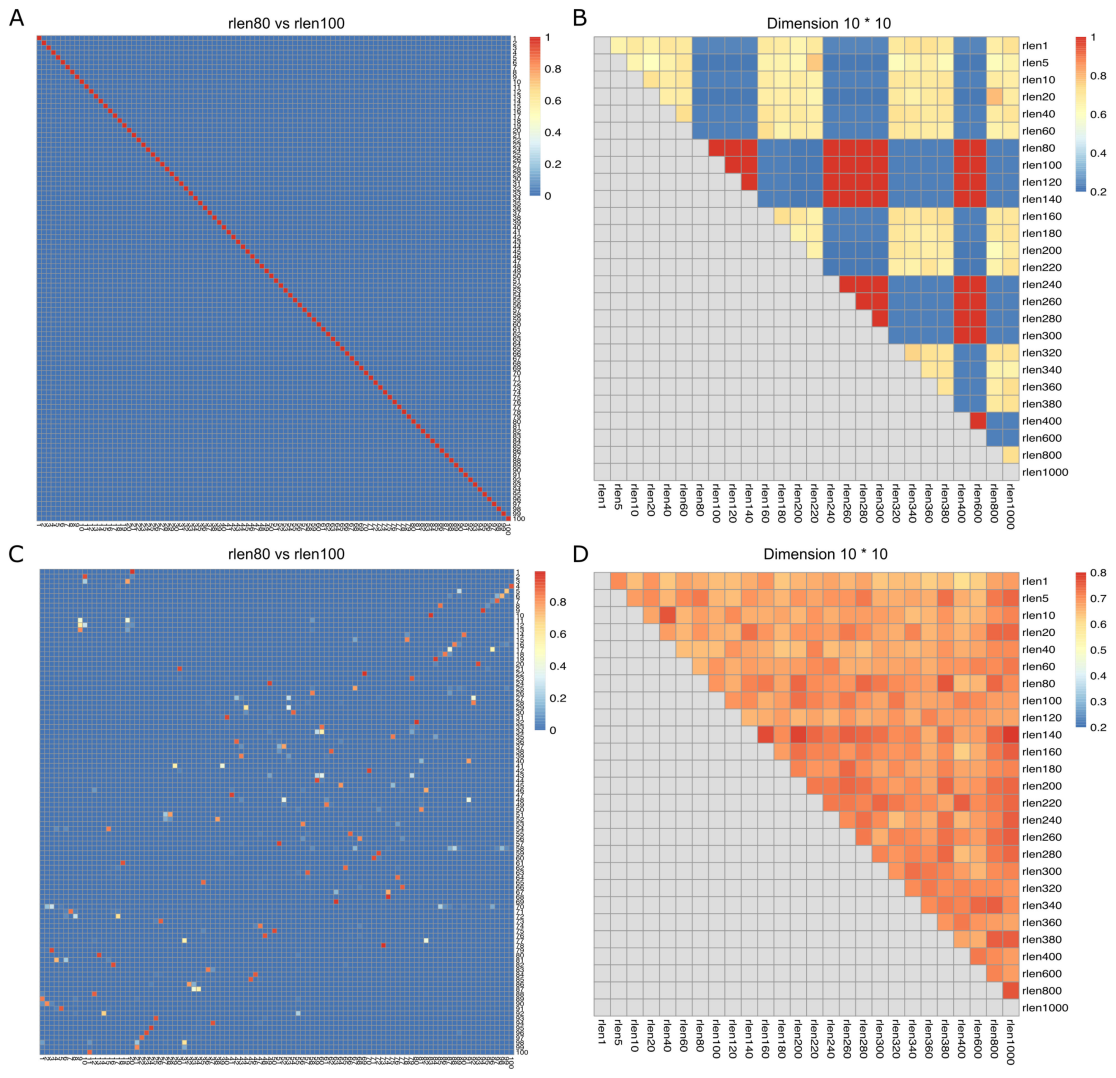
Jaccard index (degree of overlap between the cells from the two nodes) and Average Maximum Jaccard Index (AMJI) for the data set comprised of 27.5 million cells. In **(A, C)**, the heatmaps show a representative example of the Jaccard index between grid nodes from two consecutive runs before **(A)** and after **(C)** fixing the bugs in FlowSOM. As an example, the numbers on the X axis are the node indices in the run with rlen = 80, and the numbers on the Y axis are the node indices in the run with rlen = 100. The color bar indicates the scale of Jaccard index, the highest Jaccard index is in red and the lowest Jaccard index is in blue. The diagonal red line in **(A)** indicates a perfect overlap of nodes from the run with rlen = 80 and the run with rlen = 100. But in **(C)** no diagonal red line is observed. In **(B, D)**, the heatmaps show AMJI between grid nodes from any different runs before **(B)** and after **(D)** fixing the bugs in FlowSOM. Unlike the Jaccard index comparing the similarity of every pair of nodes between two different runs, AMJI is a metric summarizing the similarity of all nodes in two different runs.

The nodes within SOM persistently update and move throughout a user-specified number of iterations (rlen) during the training process. This implies that the distances of cells to their nearest node undergo continuous changes throughout the training period. We used the down-sampled dataset (see Methods) consisting of 27.5 million cells to assess how changing the FlowSOM parameters affects the clustering results. We changed the number of iterations (rlen; from 1 to 1000) and assessed the stability of the SOM (see Methods) while maintaining other parameters at their default values. The Average Distances (AD) of cells to their nearest node exhibited low values with minor fluctuations when ‘rlen’ was below ~100 indicating an updating but stable SOM. However, AD remained constant within the range of ~100 to ~140 and oscillated dramatically when ‘rlen’ exceeded ~140 (**Supplementary Figure S1A**), indicating that the nodes in SOM were not updated for some runs with ~100< rlen< ~140, which must not be observed if SOM was trained correctly. Subsequently, the Average Maximum Jaccard index (see Methods) was employed to appraise the similarity of SOMs across different runs. The analysis revealed that the SOMs from the run with ‘rlen’ values ranging from 80 to 140, and from 240 to 300, were identical, indicated by the red diagonal line (**Figures 1A, B** and **Supplementary Figure S1A**). Intriguingly, this phenomenon ceased to manifest when utilizing a smaller dataset (such as 1 million cells), with ‘rlen’ ranging from 1 to 1000 (figure not shown), implying a potential bug in the current version of FlowSOM package, rendering it incapable of analyzing datasets with tens of millions of cells.

Upon examining the source C code of FlowSOM, we uncovered a significant numerical/coding error. The data type representing the actual number of node updates (“niter”, calculated as the product of rlen and the number of cells) is hardcoded as an integer (“int”) data type. On R platforms, both 32-bit and 64-bit systems, the “int” data type in C code is typically limited to 32 bits, with a maximum value of 2,147,483,647 (2^31^-1).

When the product of rlen and the number of cells exceeds 2^31^-1, the variable “niter” in the C code is assigned a random number between -2^31^ and 2^31^-1. If this random number is positive, the SOM nodes are updated that many times after initialization during training. Conversely, if it’s negative, the SOM nodes remain un-updated after initialization. A potential solution to this bug would be replacing the “int” type by “long” or “R_xlen_t” type.

Furthermore, we observed that when analyzing a CyTOF dataset of dimensions m×n (where m is the number of channels and n is the number of cells), FlowSOM would generate an error if m×n > 2^31^-1. This issue stems from FlowSOM’s use of the “.C” function as the R interface function, which only accepts arrays up to 2^31^-1 in length. The CyTOF data must be coerced into an array of length m×n before being processed by the C code in FlowSOM for SOM training. To circumvent this limitation, we implemented the “.Call” interface to call C functions in the FlowSOM package. After addressing these bugs (see **Supplementary Figure S2** for illustration) in the FlowSOM package (version 2.6.0), no diagonal red line was observed, meaning that the nodes from two consecutive runs were not identical, suggesting that our pipeline can be correctly executed on the dataset of 27.5 million cells (**Figures 1C, D**, and **Supplementary Figure S1B**).

### 3.2 The stability of SOM is strongly dependent on grid dimension, rlens, learning rate and the size of dataset

**Figure 2:**
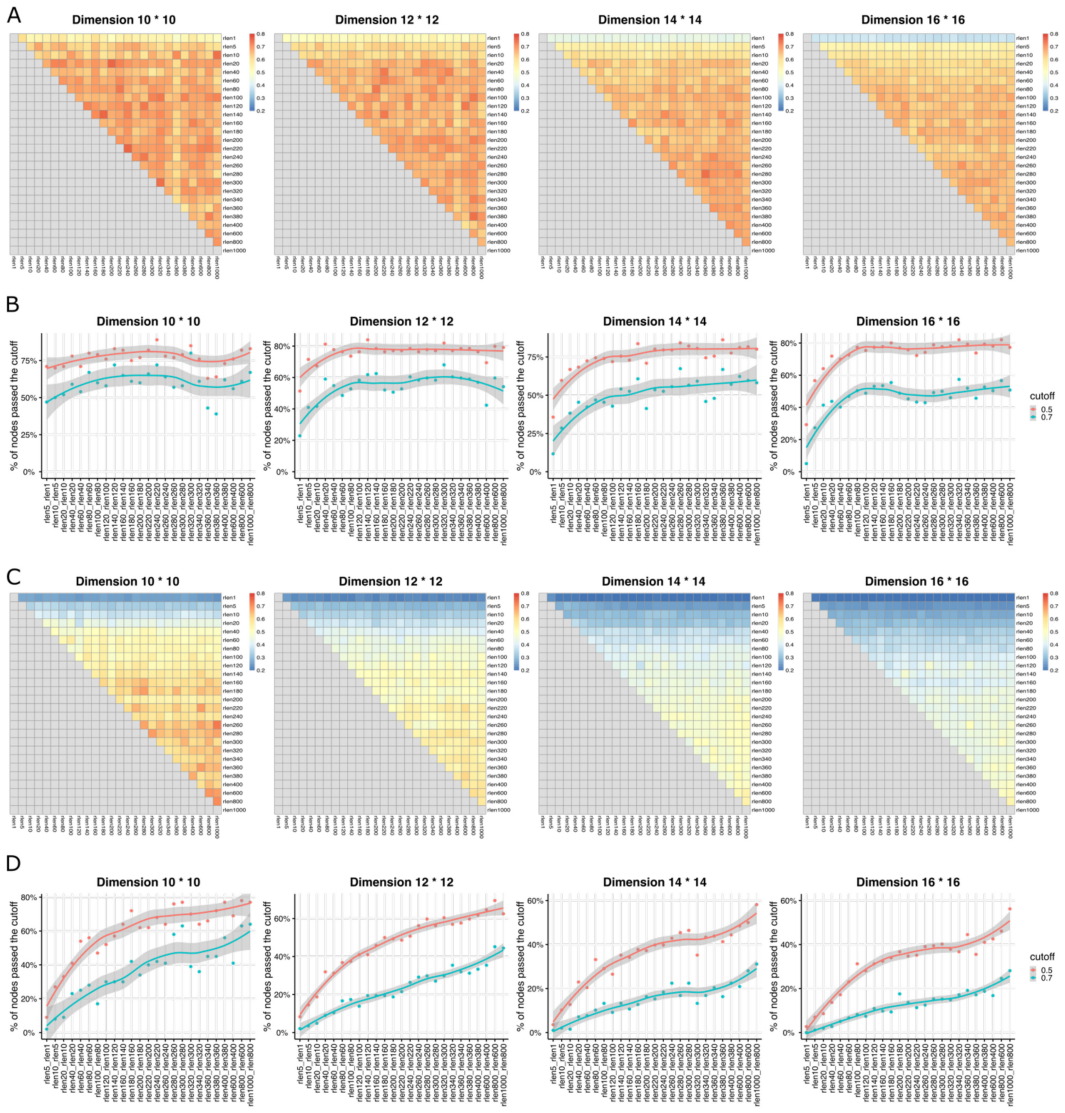
The heatmaps show Average Maximum Jaccard Index (AMJI) between different runs (with different rlen while maintaining the other parameters fixed) for the data set comprised of **(A)** 1 million cells and **(C)** 10,000 cells. In **(B, D)**, the smooth curves represent the results of a LOWESS (Locally Weighted Scatterplot Smoothing, a non-parametric regression method used to create a smooth line through a scatterplot to help visualize the underlying trend in the data) fitting applied to the Percentage of Maximum Jaccard Index Over Threshold (PMJIOT). Two consecutive runs (two consecutive rlen while maintaining the other parameters fixed) are compared for the data set comprised of **(B)** 1 million cells and **(D)** 10,000 cells. Different panels within **(A–D)** represent results with different grid dimensions while maintaining other parameters fixed.

To assess if the stability of the Self-Organizing Map (SOM) is influenced by parameters such as “rlen,” “number of cells,” “grid dimension,” and “learning rate” (**Table 1**), we used three metrics including Average Distance (AD), Average Maximum Jaccard Index (AMJI), and Percentage of Maximum Jaccard Index Over a Threshold (PMJIOT), to quantify the SOM stability between different runs. We conducted analyses with different “rlen” values while keeping other parameters constant. Using a dataset of 27.5 million cells and varying “rlen” from 1 to 1000, FlowSOM demonstrated stability with different grid dimensions (**Figures 1D**, **Supplementary Figures S1B**, **S3A, B**). However, as the dataset size decreased from 27.5 million to 100 thousand cells (and even fewer), achieving a stable SOM became challenging with small “rlen” values, evidenced by high AD (**Supplementary Figures S1B–E**), and low AMJI (**Figures 2A, C** and **Supplementary Figures S3A, C**) and PMJIOT (**Figures 2B, D** and **Supplementary Figures S3B, D**). This indicates that default parameters, like “rlen = 10” and “grid dimension = 10 × 10,” do not yield stable clusters for datasets with fewer than 100 thousand cells, rendering subsequent analyses unreliable. Moreover, when employing higher grid dimensions (12 × 12, 14 × 14, and 16 × 16), FlowSOM required even higher “rlen” to achieve stability, implying that users aiming for more detailed cell type granularity by increasing grid dimensions should adjust “rlen” accordingly, especially with smaller datasets (**Figures 2**, **Supplementary Figures S1**, **S3**).

**Figures 2A**, and **B** depicts the performance of achieving a stable SOM with varying rlen and grid dimension parameters, alongside the default learning rate of (0.05, 0.01) in FlowSOM, with a dataset containing 1 million cells. The results demonstrate that a stable SOM can be attained using default parameters (rlen = 10, grid dimension = 10 × 10) under these conditions. However, if users opt for a grid dimension of ≥ 12 × 12, the default rlen of 10 may be insufficient to achieve a stable SOM, suggesting that SOMs with higher grid dimensions required more iterations for stability under the same learning rate and dataset conditions (**Supplementary Figure S4**). Further stability exploration using different learning rates, such as (0.05, 0.001), (0.01, 0.001), and (0.1, 0.05), while keeping other parameters at default (except for rlen), revealed that a higher learning rate led to rapid SOM stability compared to a lower learning rate (**Supplementary Figure S4**). This suggests users can increase the learning rate as an alternative to rlen for achieving a stable SOM, with the caution that an excessively high learning rate may result in an unstable SOM due to large steps in grid node updates during training. Thus, it is important to perform parameter optimization to evaluate the stability of SOM for each dataset considering the number of cells, grid dimensions, rlen, and learning rate.

### 3.3 An unstable SOM could lead to biological misinterpretation

**Figure 3:**
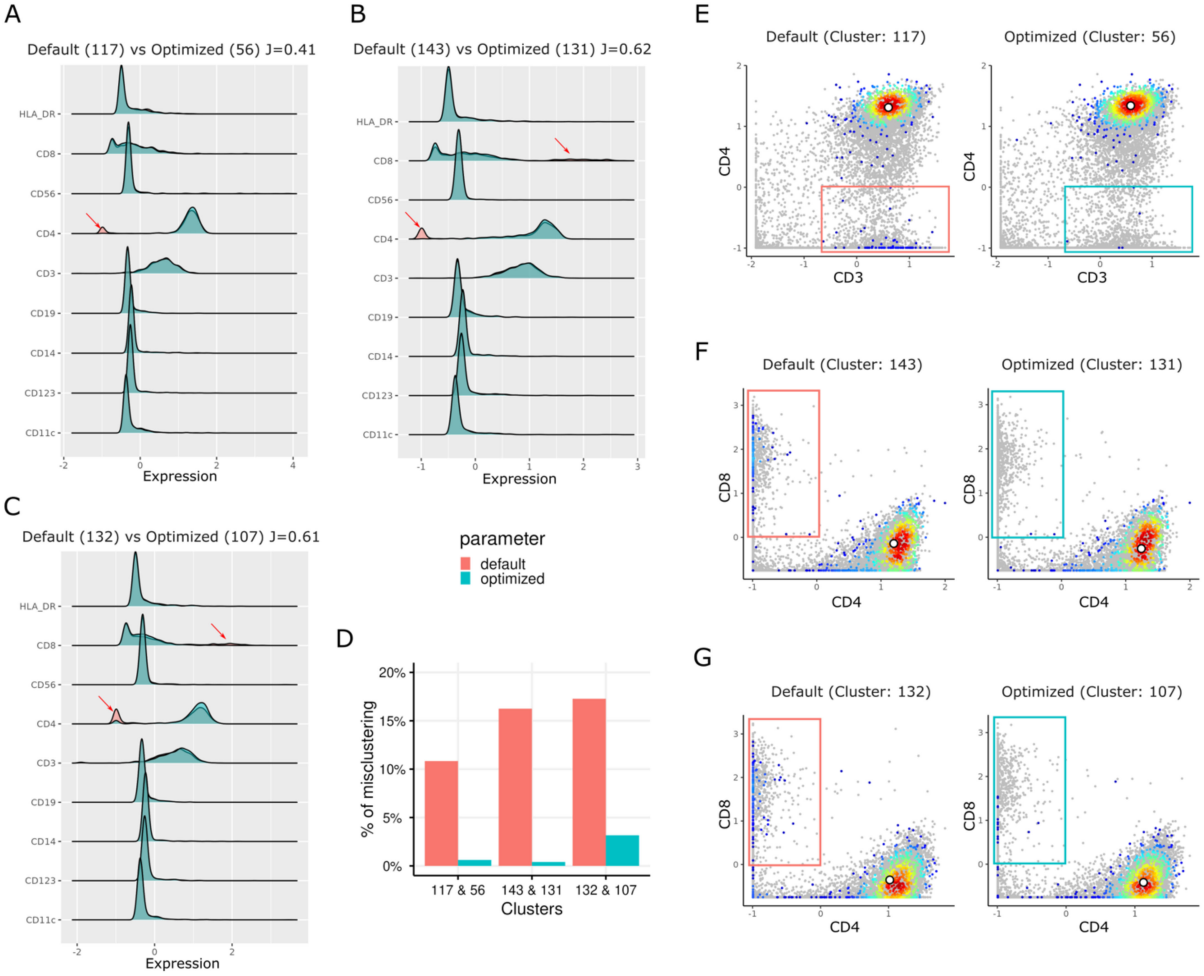
Rigid plots and biaxial plots show the different distributions of marker expression for the clusters from the default run (rlen = 10) and the optimized run (rlen = 140) when the grid dimension of 12 × 12 and data set of 100,000 cells was used. **(A, E)** are used to compare cluster 117 from default run and cluster 56 from the optimized run. Similarly, **(B, F)** are used to compare cluster 143 from default run and cluster 131 from the optimized run. **(C, G)** compare cluster 132 from default run and cluster 107 from the optimized run. **(D)** boxplot shows the percentage of misclustered cells (red and blue boxes in **E–G**) in the clusters in default and optimized runs. In **(A-C)** J denotes Jaccard index between two clusters. The grey points in E-G are the cells randomly sampled from background cells.

We used the aforementioned peanut allergy study data set, and down sampled to 100,000 cells, to determine if an unoptimized SOM could generate heterogeneous cell clusters, potentially causing biological misinterpretation. Subsequently, we calculated the Jaccard indices (**Supplementary Figure S6**) to compare clusters from two different runs: one with a grid dimension of 12 × 12 using default parameters (**Table 1** and **Supplementary Table S1**, with rlen = 10, alpha = (0.05, 0.01), etc.) and another with the same grid dimension but with an optimized rlen = 140 (cutoff at the plateau in **Supplementary Figure S3D**). Heatmaps of markers’ pseudo-bulk expression in clusters (mean expression of each marker in the cells of each cluster) for both default (**Supplementary Figure S7**) and optimized settings (**Supplementary Figure S8**) are included in Supplementary Information. We matched each cluster from the default run with the cluster from the optimized run with the maximum Jaccard index, resulting in 144 (12 × 12) pairs of clusters. For example, clusters 117, 132, and 143 from default run are annotated as CD4^+^ T cell clusters, due to their high expression of CD3 and CD4 markers, and low expression of CD14, CD11c, CD123, and CD19 markers (**Figures 3A–C**). According to the maximum Jaccard indices of these three clusters from the default run, the corresponding clusters from the optimized run are 56, 107, and 131, respectively (**Supplementary Figure S6**). However, the clusters from the default run showed a bimodal distribution of CD4 expression (**Figures 3A–C**) indicating heterogeneity in cell populations. The biaxial plots (**Figures 3E–G**) confirmed that the cells in these clusters were a mixture of CD4^+^ T cell population and CD8^+^ T cell population (or CD4^–^ T cell population). Under optimized parameters, the CD8^+^ T cells significantly decreased, resulting in more homogeneous CD4^+^ T cell fractions (**Figures 3D–G**). More specifically, after parameter optimization, cluster 131 (rlen = 140) was more homogenous than its counterpart (cluster 143 when rlen = 10) in the default settings (**Figure 3H**). Before parameter optimization, cluster 143 from the default run had ~16% of cells coming from CD8^+^ T cells, while after parameter optimization, the percentage decreased to less than 1% in its counterpart cluster 131 from optimized run (**Figures 3D, F**). These clusters (cluster 131 from optimized run or cluster 143 from default run) was annotated as a subpopulation of naive CD4^+^ T cells that play an essential role in peanut allergy as its frequency was decreased during allergic sensitization (23). Given the differences of the cell percentage changes between default and optimized runs, immunologists might formulate a wrong conclusion if the optimized parameters were not chosen. These results suggest that the parameter optimization is not only important for SOM stabilization, but also crucial for correct biological interpretation and accurate monitoring of cell population changes in health and disease.

## 4 Discussion

This study highlights the pivotal significance of parameter optimization when applying FlowSOM to clustering large biological datasets, focusing on the diverse parameters and their impact on Self-Organizing Maps (SOMs) stability. Our exploration, conducted on a public dataset comprising approximately 127 million cells, brought to light two previously unidentified bugs in FlowSOM version 2.6.0. These bugs, rooted in the use of a 32-bit integer in the backend C code, hindered the accurate analysis of datasets with a large number of cells, resulting in inconsistent SOM outputs. Following thorough debugging and rectification, our refined pipeline demonstrated the robustness of the algorithm under appropriate configurations. Its effectiveness was validated by testing it on three different internal datasets. The data supporting this validation is not included here and is currently being pursued for publication in a separate research article. This emphasizes the crucial necessity of resolving technical constraints in cytometry data analysis tools, guaranteeing their compatibility with the extensive datasets commonly encountered in biological research.

Considering widespread usage of FlowSOM across numerous datasets, each characterized by substantial cell counts ranging from hundreds of thousands to millions, it is noteworthy that FlowSOM generally functions correctly with default parameters. This recognition positions FlowSOM as a leading pipeline for the analysis of cytometry datasets. However, our investigation has brought to light a potential for the emergence of entirely unstable clusters when the product of the default “rlen” and cell counts in a dataset exceeds 2^31^ - 1. This underscores the imperative necessity for debugging the publicly available FlowSOM package. Despite its capacity to analyze smaller datasets without errors, default parameters may predispose instances of unstable clusters, thereby underscoring the importance of parameter optimization prior to executing the complete FlowSOM pipeline. Beyond debugging challenges, our investigation explored the influence of different parameters on FlowSOM’s clustering performance, scrutinizing “rlen,” “grid dimension,” “learning rate,” and “dataset size.” The FlowSOM clustering outcome is crucial for data interpretation as different parameter combinations result in distinct performances, providing evidence for optimization strategies.

The incorporation of metrics assessing clustering stability, such as the Average Maximum Jaccard Index (AMJI), yielded valuable insights into the stability and reliability of clustering outcomes. Elevated AMJI values served as indicators of more robust clustering. An assessment of AMJI scores across diverse parameter configurations facilitated the evaluation of each setting’s effectiveness. The number of training iterations denoted as “rlen” emerged as a pivotal determinant influencing clustering performance. Lower “rlen” values correlated with unstable clustering, while higher values, up to a specific threshold, contributed to enhanced consistency. The identification of the optimal number of iterations where clustering stability reached a plateau, proved essential for ensuring dependable results. Exploration of various grid dimensions allowed for further refinement of clustering output. While larger grid dimensions facilitated more detailed clustering, they concurrently heightened computational complexity and resource requirements. A comparative analysis of different grid dimensions, grounded in AMJI scores, assisted in striking a balance between the granularity of clustering and faster output time.

As one of the fundamental steps in FlowSOM, metaclustering (a step to merge similar SOM nodes/clusters into higher-level clusters, i.e., metaclusters) can merge many small cell clusters into a few relatively larger metaclusters, which can facilitate cell annotation and biological interpretation. We explored the impact of the “rlen” and “grid dimensions” on the number of metaclusters in this study as well (**Supplementary Figure S5**), given the maximum number of metaclusters being 80 and with other default parameters. On one hand, the analysis of metacluster numbers generated by FlowSOM with different rlen values for the same grid dimensions revealed an unpredictable pattern (**Supplementary Figure S5**). On the other hand, to some extent, a positive correlation was observed between the number of metaclusters and grid dimensions. Determining the appropriate number of metaclusters remains a challenge, which is subjective and varies across different datasets. One limitation of our study is that we did not address this challenge. Optimizing the parameters in FlowSOM analysis, such as rlen and grid dimensions, can profoundly impact the biological interpretation of CyTOF data. This optimization process involves a delicate balance between computational efficiency and biological accuracy. Increasing these parameters typically results in more refined and stable clusters, potentially unveiling subtle cell populations that might otherwise be overlooked. However, this comes at the cost of increased computational time. Conversely, decreasing these parameter values might lead to less distinct clusters, potentially merging biologically relevant populations and oversimplifying the data (i.e. introducing contaminating CD8^+^ T cells in cluster 143 as compared to cluster 131, which would lead to incorrect interpretation). The choice of parameters influences the resolution and stability of clusters as we have observed with CD4^+^ T cells (cluster 132 – central memory CD4^+^ T cells) which contains contaminating cells coming from the neighboring cluster 143 (activated T cells producing IFNg) that were misclassified. This issue was resolved as shown in cluster 107 using the optimized settings in FlowSOM (**Figure 3G**). This, in turn, impacts the final clustering and visualization results. Careful optimization of rlen, grid dimension, and other parameters is therefore crucial to ensure that the resulting clusters accurately represent the underlying cellular populations in the CyTOF data, striking a balance between revealing important cellular heterogeneity and avoiding oversimplifications.

Although the manuscript focuses on using one public dataset for optimization of parameters in FlowSOM, we further down sampled the dataset into 4 smaller datasets, which were used for evaluating the effect of dataset size on the SOM stability. It showed that parameter optimization is even more required for relatively smaller datasets. In addition, we have also tested our pipeline on three internal datasets (results not shown). FlowSOM parameter optimization contributed to enhancing robustness of clustering across these datasets and enabled the identification of certain rare immune cell populations. By using optimized parameters, we observed a reduction in heterogeneous clusters and their misrepresentation. This, in turn, facilitated improved annotation and biological interpretation, thereby supporting the generalizability of our approach.

In summary, optimizing parameter combinations in FlowSOM proves instrumental in identifying tailored settings for clustering biological datasets. A thorough evaluation of iterations, grid dimension, and learning rate ensures reliable and meaningful clusters, emphasizing clustering stability. FlowSOM emerges as an automated, data-driven approach for high-dimensional cell phenotyping, making a significant contribution to the ongoing discourse on CyTOF/Flow data analysis. From debugging technical limitations to unraveling intricate parameter dependencies, our findings underscore the need for vigilant algorithmic development and verification, emphasizing the importance of understanding parameter interactions for robust and reproducible cytometry data analysis. While the debugged version of FlowSOM can effectively handle large datasets exceeding 50 million cells with default parameter settings, datasets with fewer than 1 million cells require careful parameter optimization to achieve stable and reliable clustering.

## Data Availability

The original contributions presented in the study are included in the article/Supplementary Material, further inquiries can be directed to the corresponding author/s.

## Footnotes

1 available at http://flowrepository.org/id/FR-FCM-Z4MA

## Supplementary material

Supplementary Figure S1 (A) The line plots represent Average Distance (AD) of cells to the nearest node in different runs with different rlen for the dataset comprised of 27.5 million cells using FlowSOM before fixing the bugs. Different panels in **(A)** represent different grid dimensions, while maintaining other parameters fixed, shown on the top of each panel. After fixing the bugs in FlowSOM, Average Distance of cells to the nearest node is similarly shown in **(B–E)**, which are the plots for the datasets comprised of 27.5 million, 1 million, 100,000, and 10,000 cells, respectively.

Supplementary Figure S2 The illustration of changes in the debugged version of FlowSOM package. Red color highlights the earlier version of the code, and the blue highlights the debugged version. The code in the orange box is C code.

Supplementary Figure S3 The heatmaps show the Average Maximum Jaccard Index (AMJI) for the data set comprised of **(A)** 27.5 million cells and **(C)** 100,000 cells. In **(B, D)**, the smooth curves represent the results of a LOWESS (Locally Weighted Scatterplot Smoothing) fitting applied to the Percentage of Maximum Jaccard Index Over Threshold (PMJIOT). Two consecutive runs (two consecutive rlen while maintaining the other parameters fixed) are compared for the data set comprised of (B) 27.5 million cells and **(D)** 100,000 cells. Different panels within **(A–D)** represent results with different grid dimensions while maintaining other parameters fixed.

Supplementary Figure S4 The smooth curves represent the results of a LOWESS (Locally Weighted Scatterplot Smoothing) fitting applied to the Percentage of Maximum Jaccard Index Over Threshold (PMJIOT) in the dataset comprised of 1 million cells using **(A)** learning rate = (0.05, 0.001), **(B)** learning rate = (0.01, 0.001), and **(C)** learning rate = (0.1, 0.05), while maintaining other parameters fixed. Different colors represent different thresholds in the PMJIOT formula in the Methods. Different panels within **(A–C)** represent results with different grid dimensions while maintaining other parameters fixed.

Supplementary Figure S5 The number of metaclusters varies in different runs given different grid dimensions (10, 12, 14, and 16 represented in different colors), while maintaining other parameters fixed for a data set comprised of **(A)** 27.5 million cells, **(B)** 1 million cells, **(C)** 100,000 cells, and **(D)** 10,000 cells.

Supplementary Figure S6 Jaccard index between the nodes from the run with default parameters (rlen = 10) and the nodes from the run with optimized rlen (140) under grid dimension = 12 × 12 setting.

Supplementary Figure S7 Heatmap showing markers’ pseudo-bulk expression in clusters (mean expression of each marker in the cells of each cluster) with the default parameter setting (rlen = 10). The color scale indicates median cluster expression ranged from blue (lowest) to red (highest).

Supplementary Figure S8 Heatmap showing markers’ pseudo-bulk expression in clusters (mean expression of each marker in the cells of each cluster) with the optimized parameter setting (rlen = 140). The color scale indicates median cluster expression ranged from blue (lowest) to red (highest).

Supplementary Table S1 The parameters that remained at default setting.

## References

[1] Abdelaal T van Unen V Höllt T Koning F Reinders MJT Mahfouz A. Predicting cell populations in single cell mass cytometry data. Cytometry A. (2019) 95:769–81. doi: 10.1002/cyto.a.23738 PMC6767556 30861637

[2] Gadalla R Noamani B MacLeod BL Dickson RJ Guo M Xu W. Validation of cyTOF against flow cytometry for immunological studies and monitoring of human cancer clinical trials. Front Oncol. (2019) 9:415. doi: 10.3389/fonc.2019.00415 31165047 PMC6534060

[3] Iyer A Hamers AAJ Pillai AB. CyTOF^®^ for the masses. Front Immunol. (2022) 13:815828. doi: 10.3389/fimmu.2022.815828 35493491 PMC9047695

[4] Korin B Dubovik T Rolls A. Mass cytometry analysis of immune cells in the brain. Nat Protoc. (2018) 13:377–91. doi: 10.1038/nprot.2017.155 29370157

[5] Bandyopadhyay S Fisher DAC Malkova O Oh ST. Analysis of signaling networks at the single-cell level using mass cytometry. Methods Mol Biol. (2017) 1636:371–92. doi: 10.1007/978-1-4939-7154-1_24 28730492

[6] Astle JM Huang H. Mass cytometry in hematologic Malignancies: research highlights and potential clinical applications. Front Oncol. (2021) 11:704464. doi: 10.3389/fonc.2021.704464 34858804 PMC8630615

[7] Liu X Song W Wong BY Zhang T Yu S Lin GN. A comparison framework and guideline of clustering methods for mass cytometry data. Genome Biol. (2019) 20:297. doi: 10.1186/s13059-019-1917-7 31870419 PMC6929440

[8] den Braanker H Bongenaar M Lubberts E. How to prepare spectral flow cytometry datasets for high dimensional data analysis: A practical workflow. Front Immunol. (2021) 12:768113. doi: 10.3389/fimmu.2021.768113 34868024 PMC8640183

[9] Nowicka M Krieg C Crowell HL Weber LM Hartmann FJ Guglietta S. CyTOF workflow: differential discovery in high-throughput high-dimensional cytometry datasets. F1000Res. (2017) 6:748. doi: 10.12688/f1000research.11622.3 28663787 PMC5473464

[10] Crowell H. CATALYST: Cytometry dATa anALYSis Tools. In: R package version 1260. Available online at: https://bioconductor.org/packages/CATALYST.

[11] Kimball AK Oko LM Bullock BL Nemenoff RA van Dyk LF Clambey ET. A beginner’s guide to analyzing and visualizing mass cytometry data. J Immunol. (2018) 200:3–22. doi: 10.4049/jimmunol.1701494 29255085 PMC5765874

[12] Levine JH Simonds EF Bendall SC Davis KL Amir ED Tadmor MD. Data-driven phenotypic dissection of AML reveals progenitor-like cells that correlate with prognosis. Cell. (2015) 162:184–97. doi: 10.1016/j.cell.2015.05.047 PMC4508757 26095251

[13] Amir ED Davis KL Tadmor MD Simonds EF Levine JH Bendall SC. viSNE enables visualization of high dimensional single-cell data and reveals phenotypic heterogeneity of leukemia. Nat Biotechnol. (2013) 31:545–52. doi: 10.1038/nbt.2594 PMC4076922 23685480

[14] Qiu P Simonds EF Bendall SC Gibbs KD Bruggner RV Linderman MD. Extracting a cellular hierarchy from high-dimensional cytometry data with SPADE. Nat Biotechnol. (2011) 29:886–91. doi: 10.1038/nbt.1991 PMC3196363 21964415

[15] Van Gassen S Callebaut B Van Helden MJ Lambrecht BN Demeester P Dhaene T. FlowSOM: Using self-organizing maps for visualization and interpretation of cytometry data. Cytometry A. (2015) 87:636–45. doi: 10.1002/cyto.a.22625 25573116

[16] Quintelier K Couckuyt A Emmaneel A Aerts J Saeys Y Van Gassen S. Analyzing high-dimensional cytometry data using FlowSOM. Nat Protoc. (2021) 16:3775–801. doi: 10.1038/s41596-021-00550-0 34172973

[17] Weber LM Robinson MD. Comparison of clustering methods for high-dimensional single-cell flow and mass cytometry data. Cytometry A. (2016) 89:1084–96. doi: 10.1002/cyto.a.23030 27992111

[18] Wehrens R Buydens LMC. Self- and super-organizing maps in R: the kohonen package. J Stat Software. (2007). 21 (5):1–19. doi: 10.18637/jss.v021.i05

[19] Romero-Olmedo AJ Schulz AR Huber M Brehm CU Chang H-D Chiarolla CM. Deep phenotypical characterization of human CD3+ CD56+ T cells by mass cytometry. Eur J Immunol. (2021) 51:672–81. doi: 10.1002/eji.202048941 33231295

[20] Hiddingh S Pandit A Verhagen F Rijken R Servaas NH Wichers RCGK. Transcriptome network analysis implicates CX3CR1-positive type 3 dendritic cells in non-infectious uveitis. Elife. (2023) 12:e74913. doi: 10.7554/eLife.74913 37042831 PMC10185339

[21] Verhagen FH Hiddingh S Rijken R Pandit A Leijten E Olde Nordkamp M. High-dimensional profiling reveals heterogeneity of the th17 subset and its association with systemic immunomodulatory treatment in non-infectious uveitis. Front Immunol. (2018) 9:2519. doi: 10.3389/fimmu.2018.02519 30429855 PMC6220365

[22] Kohonen T. Self-organized formation of topologically correct feature maps. Biol Cybern. (1982) 43:59–69. doi: 10.1007/BF00337288

[23] Kohonen T. Essentials of the self-organizing map. Neural Networks. (2013) 37:52–65. doi: 10.1016/j.neunet.2012.09.018 23067803

[24] Kaushik A Dunham D Han X Do E Andorf S Gupta S. CD8+ T cell differentiation status correlates with the feasibility of sustained unresponsiveness following oral immunotherapy. Nat Commun. (2022) 13:6646. doi: 10.1038/s41467-022-34222-8 36333296 PMC9636180

