# Supplementary material for: Parameter optimization for stable clustering using FlowSOM: a case study from CyTOF
Source: Front Immunol. 2024 Oct 9;15:1414400. doi: 10.3389/fimmu.2024.1414400 (PMC11497637; doi:10.3389/fimmu.2024.1414400)
Supplement: Supplementary Figure S1 — (A) The line plots represent Average Distance (AD) of cells to the nearest node in different runs with different rlen for the dataset comprised of 27.5 million cells using FlowSOM before fixing the bugs. Different panels in (A) represent different grid dimensions, while maintaining other parameters fixed, shown on the top of each panel. After fixing the bugs in FlowSOM, Average Distance of cells to the nearest node is similarly shown in (B–E), which are the plots for the datasets comprised of 27.5 million, 1 million, 100,000, and 10,000 cells, respectively. [file DataSheet1.docx]

**Supplementary Information**


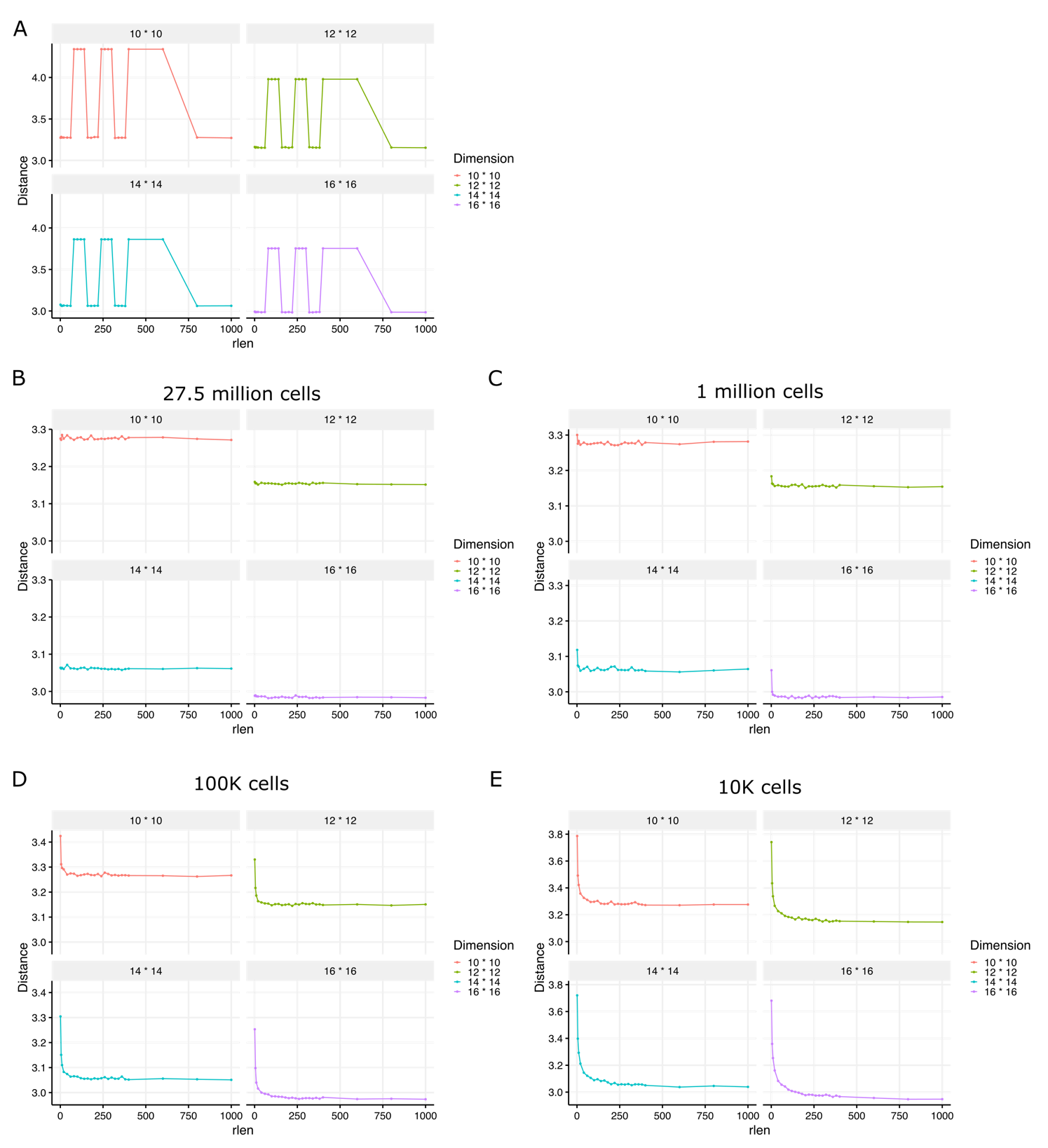


*Figure S1. (A) The line plots represent Average Distance (AD) of cells to the nearest node in different runs with different rlen for the dataset comprised of 27.5 million cells using FlowSOM before fixing the bugs. Different panels in (A) represent different grid dimensions, while maintaining other parameters fixed, shown on the top of each panel. After fixing the bugs in FlowSOM, Average Distance of cells to the nearest node is similarly shown in (B), (C), (D), and (E), which are the plots for the datasets comprised of 27.5 million, 1 million, 100,000, and 10,000 cells, respectively.*


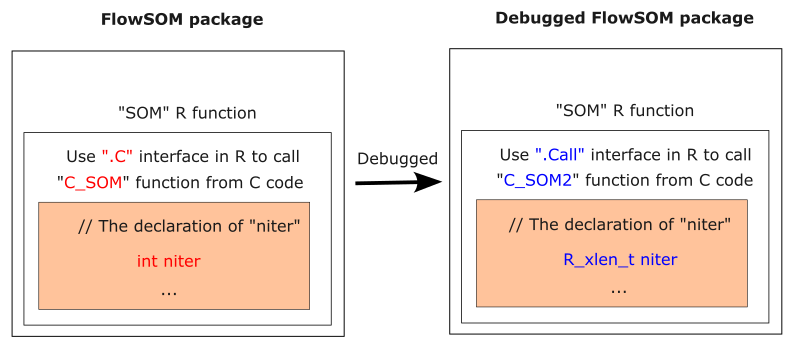


*Figure S2. The illustration of changes in the debugged version of FlowSOM package. Red color highlights the earlier version of the code, and the blue highlights the debugged version. The code in the orange box is C code.*


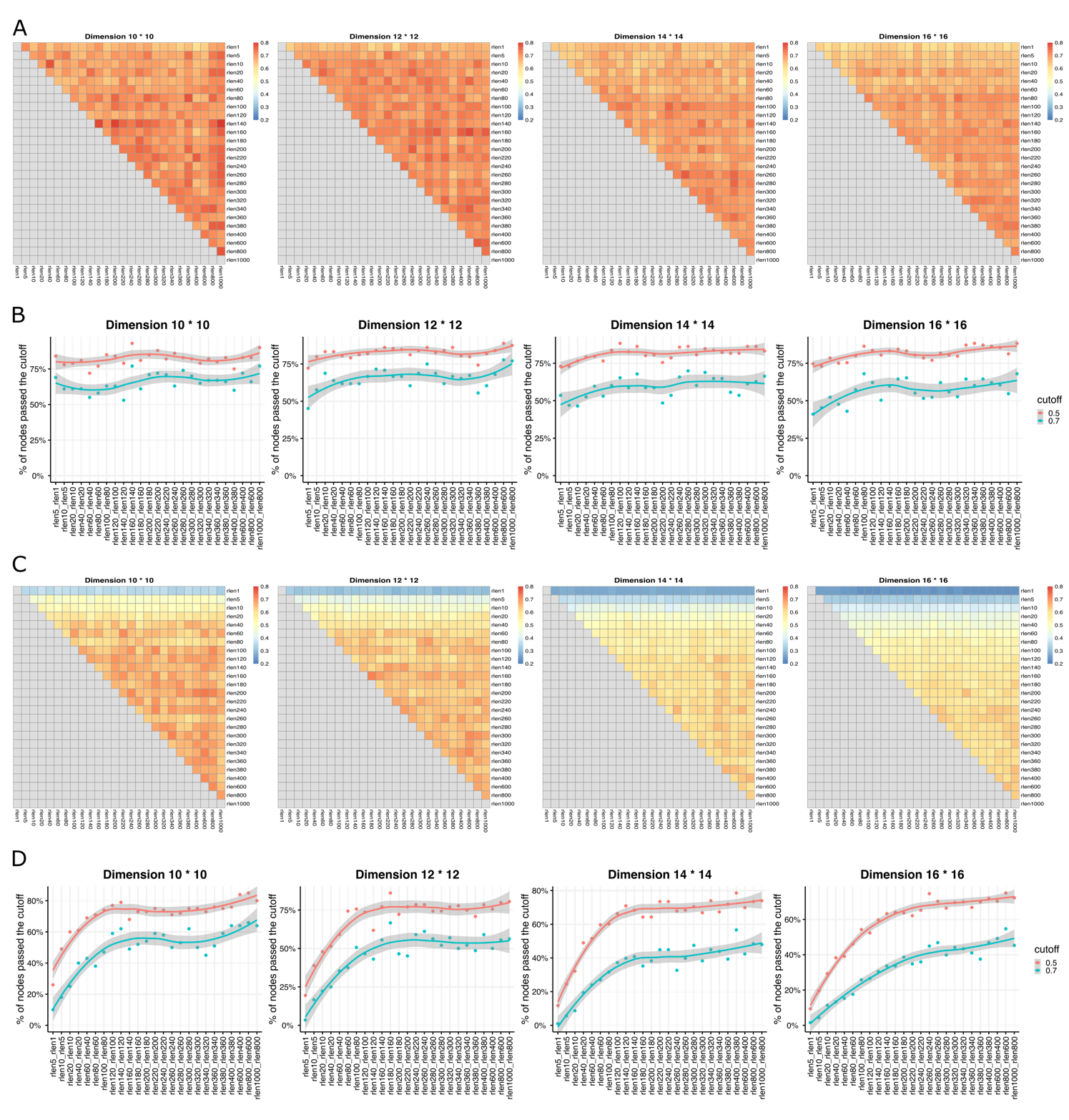


*Figure S3. The heatmaps show the Average Maximum Jaccard Index (AMJI) for the data set comprised of (A) 27.5 million cells and (C) 100,000 cells. In (B) and (D), the smooth curves represent the results of a LOWESS (Locally Weighted Scatterplot Smoothing) fitting applied to the Percentage of Maximum Jaccard Index Over Threshold (PMJIOT). Two consecutive runs (two consecutive rlen while maintaining the other parameters fixed) are compared for the data set comprised of (B) 27.5 million cells and (D) 100,000 cells. Different panels within (A), (B), (C), and (D) represent results with different grid dimensions while maintaining other parameters fixed.*


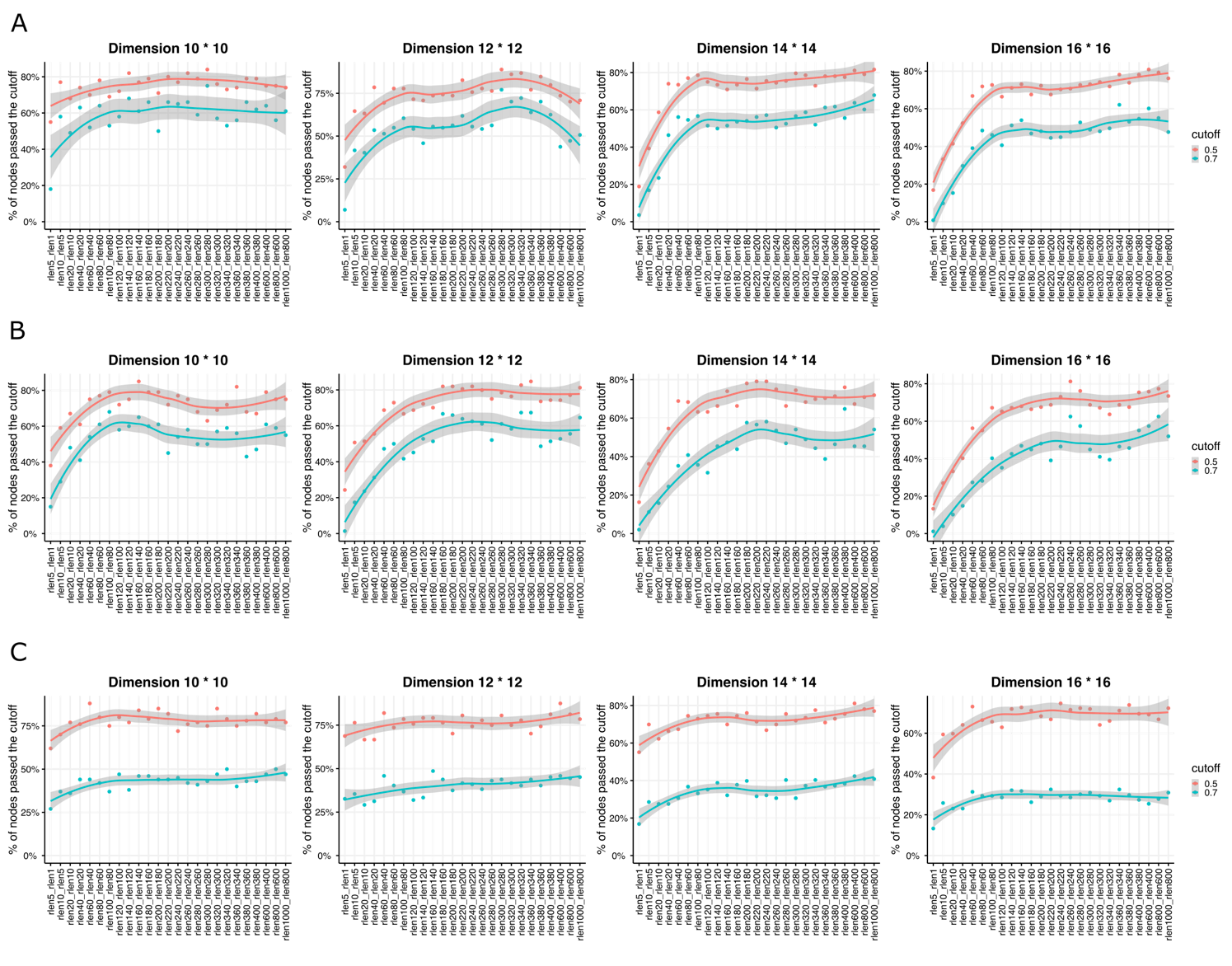


*Figure S4. The smooth curves represent the results of a LOWESS (Locally Weighted Scatterplot Smoothing) fitting applied to the Percentage of Maximum Jaccard Index Over Threshold (PMJIOT) in the dataset comprised of 1 million cells using (A) learning rate = (0.05, 0.001), (B) learning rate = (0.01, 0.001), and (C) learning rate = (0.1, 0.05), while maintaining other parameters fixed. Different colors represent different thresholds in the PMJIOT formula in the Methods. Different panels within (A), (B), and (C) represent results with different grid dimensions while maintaining other parameters fixed.*


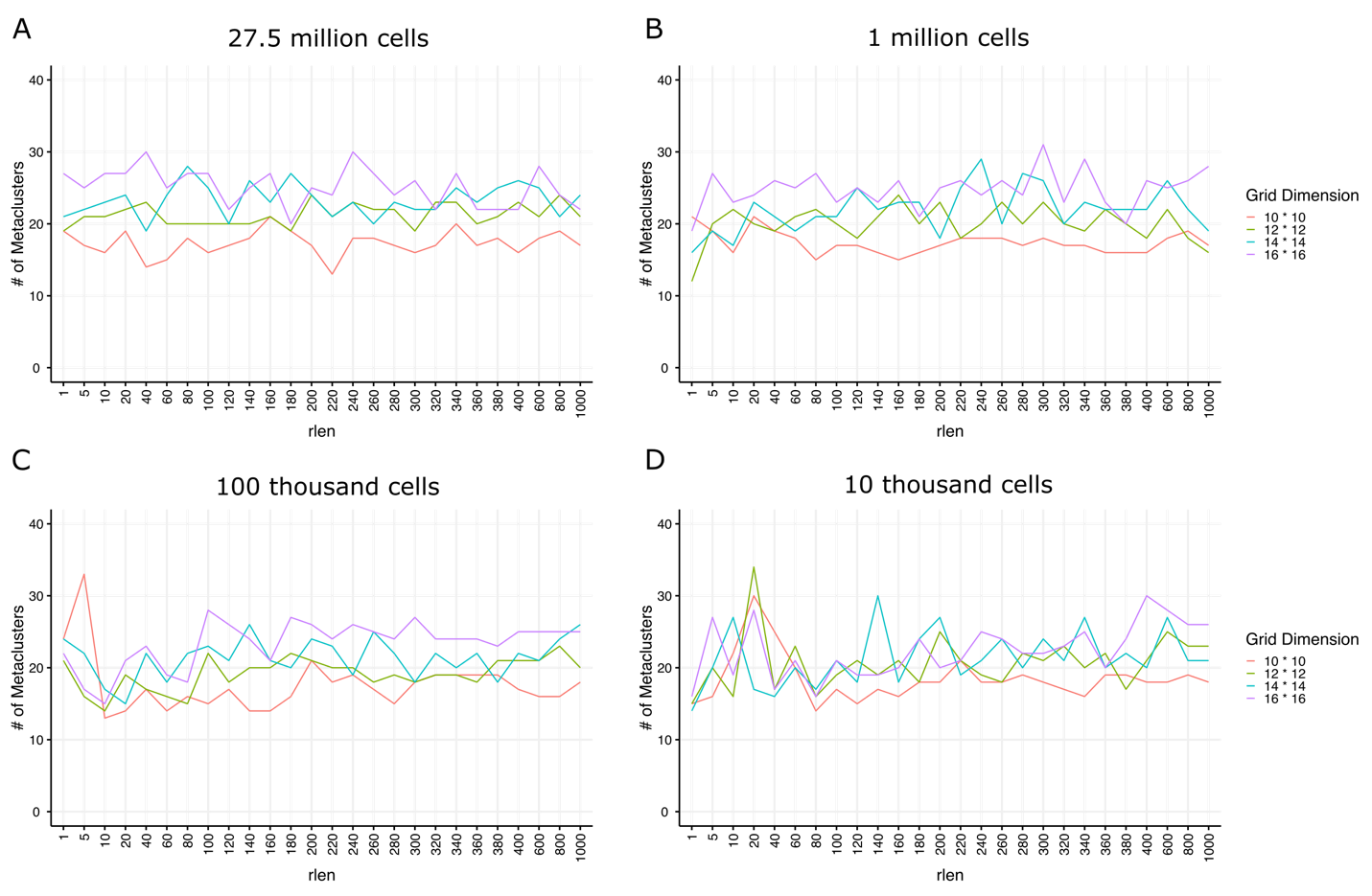


*Figure S5. The number of metaclusters varies in different runs given different grid dimensions (10, 12, 14, and 16 represented in different colors), while maintaining other parameters fixed for a data set comprised of (A) 27.5 million cells, (B) 1 million cells, (C) 100,000 cells, and (D) 10,000 cells.*


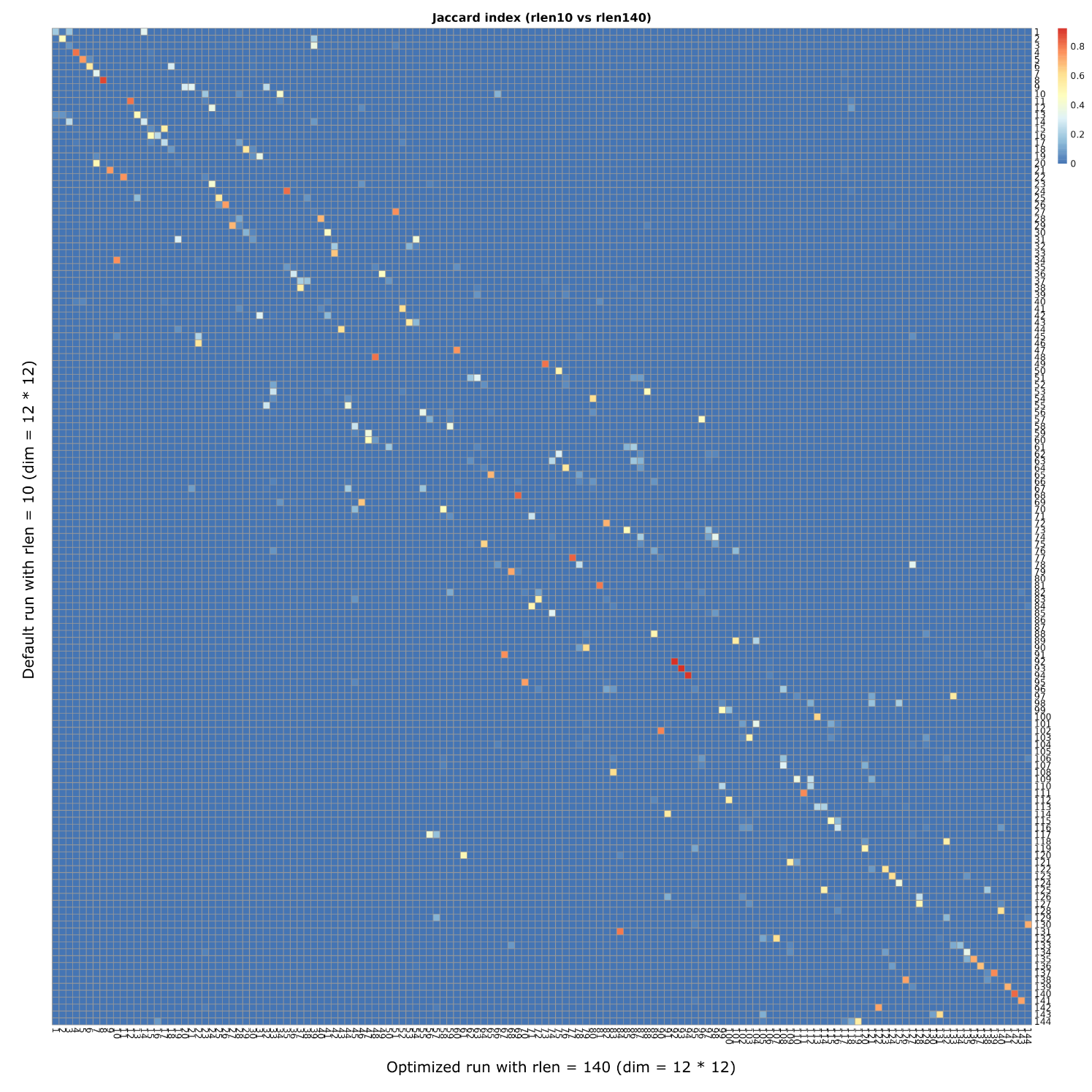


*Figure S6. Jaccard index between the nodes from the run with default parameters (rlen = 10) and the nodes from the run with optimized rlen (140) under grid dimension = 12* × *12 setting.*


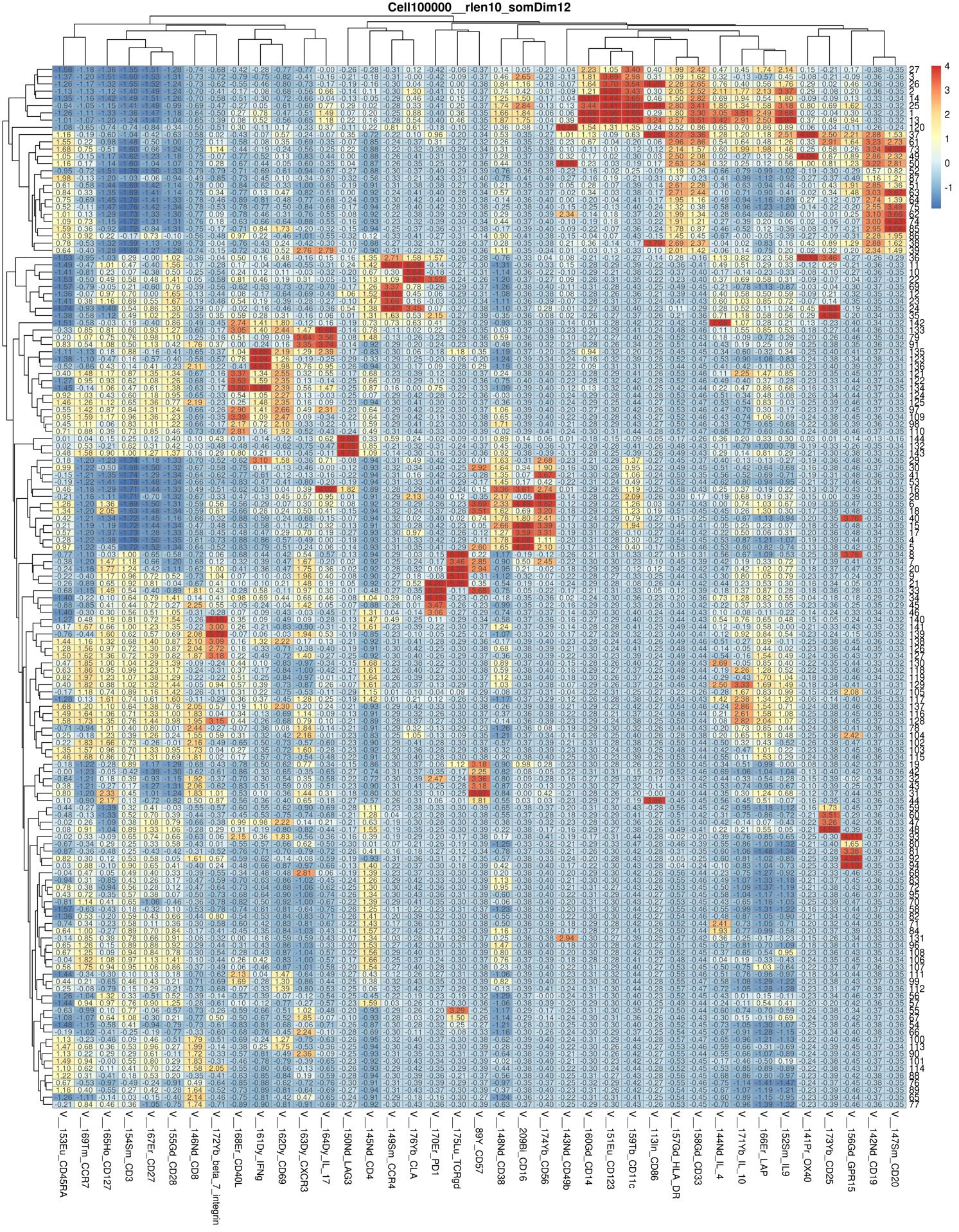
*Figure S7. Heatmap showing* markers’ pseudo-bulk expression in clusters (mean expression of each marker in the cells of each cluster) *with the default parameter setting (rlen = 10). The color scale indicates median cluster expression ranged from blue (lowest) to red (highest).*


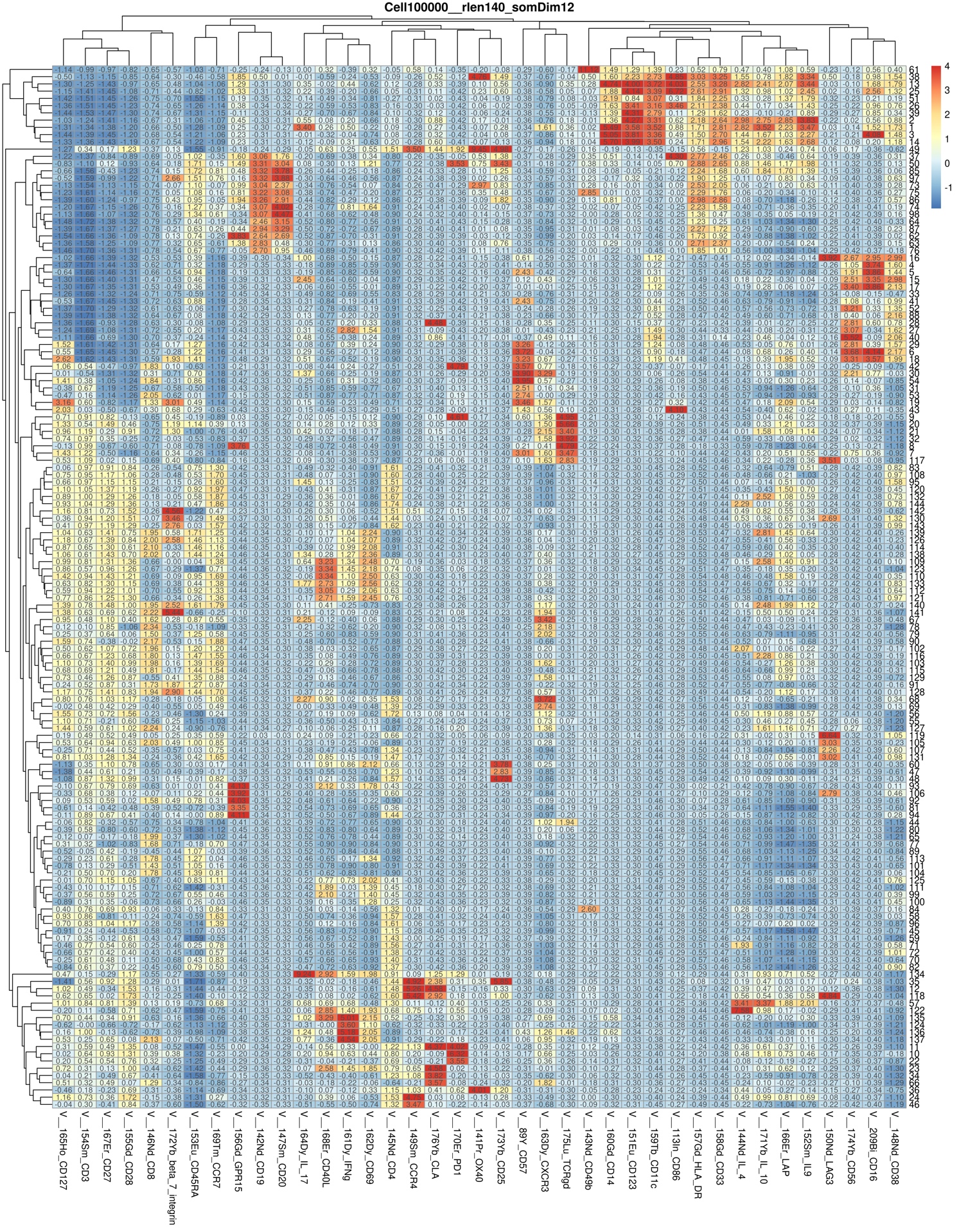


*Figure S8. Heatmap showing* markers’ pseudo-bulk expression in clusters (mean expression of each marker in the cells of each cluster) *with the optimized parameter setting (rlen = 140). The color scale indicates median cluster expression ranged from blue (lowest) to red (highest).*

Table S1. The parameters that remained at default setting.

| **Parameter** | **Explanation** | **Values** |
| --- | --- | --- |
| mst | Number of times to build a minimum spanning tree for visualization | 1 |
| radius | Start and end radius | quantile (nhbrdist, 0.67) * c(1, 0) |
| init | Whether to initialize cluster centers in a non-random way | FALSE |
| initf | Use the given initialization function if init is TRUE | Initialize_KWSP |
| distf | Distance function (1 = manhattan, 2 = euclidean, 3 = chebyshev, 4 = cosine) | 2 |
| silent | Whether to print status updates | FALSE |
| map | Whether the data is not mapped to the SOM. | TRUE |
| codes | Cluster centers to start with | NULL |
| importance | Array with numeric values. Parameters will be scaled according to importance | NULL |
